# Evaluation of the Physical Properties of Bedding Materials for Dairy Cattle Using Fuzzy Clustering Analysis

**DOI:** 10.3390/ani10020351

**Published:** 2020-02-22

**Authors:** Patrícia Ferreira Ponciano Ferraz, Gabriel Araújo e Silva Ferraz, Lorenzo Leso, Marija Klopčič, Giuseppe Rossi, Matteo Barbari

**Affiliations:** 1 Department of Agricultural Engineering, Federal University of Lavras (UFLA), Lavras, Minas Gerais 37200–900, Brazil; gabriel.ferraz@ufla.br; 2 Department of Agriculture, Food, Environment and Forestry, University of Florence, Via San Bonaventura, 13-50145 Florence, Italy; lorenzo.leso@unifi.it (L.L.); giuseppe.rossi@unifi.it (G.R.); matteo.barbari@unifi.it (M.B.); 3 Department of Animal Science, Biotechnical Faculty, University of Ljubljana, Groblje 3, 1230 Domžale, Slovenia; Marija.Klopcic@bf.uni-lj.si

**Keywords:** alternative bedding material, cattle housing systems, clustering algorithm, Gustafson–Kessel, dairy cows, water holding capacity

## Abstract

**Simple Summary:**

The bedding material used in dairy cow housing systems plays a key role in animal welfare and performance, since it influences the time that the animals remain lying down. The primary aim of this paper was to evaluate the physical properties of different bedding materials for dairy cattle and, further, to employ different fuzzy clustering algorithms to effectively cluster these alternative materials based on their physical properties. To perform nine physical analyses, 51 different bedding materials from various places in Europe were used. These data were analysed by principal components analysis (PCA) and then by fuzzy clustering analysis. Three clustering algorithms were tested for different numbers of clusters (2–8). They were compared by five validation indexes to choose the best clustering algorithm and the number of clusters. By these analyses it was possible to conclude that alternative materials can be classified based on their physical properties. The Gustafson–Kessel (GK) clustering algorithms, with eight clusters, fit better regarding the division of materials according to their properties. *Posidonia oceanica* showed potential to be used as an alternative bedding material due to its favourable physical properties.

**Abstract:**

The bedding materials used in dairy cow housing systems are extremely important for animal welfare and performance. A wide range of materials can be used as bedding for dairy cattle, but their physical properties must be analysed to evaluate their potential. In the present study, the physical properties of various bedding materials for dairy cattle were investigated, and different fuzzy clustering algorithms were employed to cluster these materials based on their physical properties. A total of 51 different bedding materials from various places in Europe were collected and tested. Physical analyses were carried out for the following parameters: bulk density (BD), water holding capacity (WHC), air-filled porosity (AFP), global density (GD), container capacity (CC), total effective porosity (TEP), saturated humidity (SH), humidity (H), and average particle size (APS). These data were analysed by principal components analysis (PCA) to reduce the amount of data and, subsequently, by fuzzy clustering analysis. Three clustering algorithms were tested: k-means (KM), fuzzy c-means (FCM) and Gustafson–Kessel (GK) algorithms. Furthermore, different numbers of clusters (2−8) were evaluated and subsequently compared using five validation indexes. The GK clustering algorithm with eight clusters fit better regarding the division of materials according to their properties. From this clustering analysis, it was possible to understand how the physical properties of the bedding materials may influence their behaviour. Among the materials that fit better as bedding materials for dairy cows, *Posidonia oceanica* (Cluster 6) can be considered an alternative material.

## 1. Introduction

The bedding material used in barns for dairy cows plays a key role in animal welfare and performance, since it influences the time that the animals remain lying down, and consequently, the processes of rumination and milk production [1,2,3]. Dairy cows can spend approximately 8−16 h/d lying down, which makes the quality of the surface important [4]. Previous studies have shown that increasing lying times are beneficial for milk production [5]; hence, it is important to provide a reasonably clean, dry, and comfortable surface for cows to rest on [6].

In addition, bedding can affect dairy cows in several ways, including comfort [2,7], cleanliness [8], behaviour [9,10], leg and hoof injuries [11,12], and health [13]. It is undoubtedly extremely important to provide dairy cattle with a good opportunity to lie, and the above mentioned studies assume that the longer the lying time, the better the welfare.

In freestall barns, the most commonly used bedding materials are sand and straw, which are used on dairy cattle farms worldwide [2]. In compost-bedded pack barns, wood shavings and sawdust are the most popular materials; however, straw, woodchips, and compost are also used [14,15,16,17].

Even though farmers are aware of the importance of bedding to the comfort of cows, these traditional bedding materials may not be available during certain seasons or in certain geographic regions; therefore, alternative bedding materials should be considered. This issue encourages farmers to search for alternative bedding materials. Alternative materials have become attractive sources of bedding, since they can be obtained locally at a low cost [18].

To propose the use and handling of an alternative bedding material, it is important to have a complete understanding of the nature of the materials involved and how their physical characteristics affect the system [19]. In the literature, it is possible to find many methodologies for the evaluation of the physical properties of bedding materials [19,20,21]; however, a limited number of studies have focused on dairy cows. Therefore, it is important to propose adaptations to these methodologies for the evaluation of the physical properties of alternative materials and their potential use as cattle bedding. According to [19], the accurate evaluation of bedding properties is important for estimating handling, storage, treatment, disposal and cost.

The present paper describes techniques for the determination of the following physical properties of bedding materials: bulk density (BD), water holding capacity (WHC), air-filled porosity (AFP), global density (GD), container capacity (CC), total effective porosity (TEP), saturated humidity (SH), humidity (H), and average particle size (APS). However, these physical properties have an impact on each another and should not be evaluated separately. In this case, the clustering technique of data mining can be a favourable unsupervised form of data classification. This clustering methodology divides the data elements into a number of groups (clusters), in order that elements within a group possess high similarity but differ from elements in other groups [22]. The goal of the fuzzy classification model proposed in the present paper was to determine boundary functions capable of performing a mapping between data samples of the physical properties of bedding materials and their classes. In essence, the classifiers attempted to find similarities between data samples, so that the distance between members of a cluster is minimised and that the distance between members of different groups is maximised [23]. The resulting classification model allowed separation of the classes of bedding materials with different physical properties.

The aim of the present study was to evaluate the physical properties of various bedding materials for dairy cattle, and further employ different fuzzy clustering algorithms to effectively cluster these alternative materials based on their physical properties.

## 2. Materials and Methods

### 2.1. Physical Properties of the Bedding Materials

The experiment was carried out in the Department of Agriculture, Food, Environment and Forestry (DAGRI) at the University of Florence. Table 1 shows the 51 different materials obtained from farms in three different European countries. The present study was part of the EU-funded project, “FreeWalk: Develop economic sound free walk farming systems elevating animal welfare, health and manure quality, while being appreciated by society” (ERA-NET SusAn program). Physical analysis was carried out for the following parameters: bulk density (BD), water holding capacity (WHC), air-filled porosity (AFP), global density (GD), container capacity (CC), total effective porosity (TEP), saturated humidity (SH), humidity (H), and average particle size (APS).

The BD of the materials was determined according to the American Society of Agricultural Engineers—ASAE Standard S269.4 DEC 91 [24]. Three cylindrical glass containers were used, each with a specific internal diameter (DT). The tested bedding material was poured into the container from a certain height to facilitate the free flow of the samples until the container was overflowing. The excess material was removed by striking a straight edge across the top. The weight of the material and the container was recorded, and the net weight of the sample was obtained by subtracting the weight of the empty container. The BD (kg m^−3^) was calculated using Equation (1):

(1) $$BD=\frac{M}{V}$$

where M is the mass of the tested bedding material (kg) and V is the container volume (m^3^).

To obtain the WHC, AFP, GD, CC, and TEP, the methodology described by [25] was adapted in accordance with the AS 3743–2003 (Appendix B Method) [26]. According to [25], the analysis was performed using two pieces of PVC tube (internal diameter, 8.7 cm; length, 12.0 cm); one capped on the bottom with four holes and the second adapted so that it fit snugly over the top of the first piece (bottom tube and top tube, respectively). The volume of the bottom tube was calibrated by filling the tube with water and gravimetrically determining the volume added.

Each tested bedding material was poured into the top of the tube (both pieces had been joined together by this stage) until the top section was at least half full. The apparatus was soaked three times in a container of water, so that the entire material sample was completely submerged. The top section of tube was carefully removed, and the surface of the material was levelled in the bottom tube. The bottom tube was lowered into water until the water was level with the top surface of the material and the tube. The holes were blocked as the apparatus was removed from the water. The water was drained for up to 60 min into a pre-weighed container. The entire saturated sample was subsequently poured into a pre-weighed sample dish and dried at 65 °C until it reached a stable weight. The WHC was calculated in kg.kg^−1^ using Equation (2):

(2) $$WHC=\frac{\left(M_{w}-M_{d}\right)}{V_{b}}\times BD$$

where M_w_ is the mass of the saturated material in the bottom tube (kg), M_d_ is the oven dried mass of the material in the bottom tube (Kg), V_b_ is the volume of the bottom tube (m³), and BD is the bulk density (kg m^−3^).

The CC is the volume of water retained in the bedding material following saturation and free drainage without evaporation [27]. According to [28], the CC is the maximum water retention capacity of a material in a given container under the same conditions of saturation and drainage. The CC (%) can be calculated by Equation (3):

(3) $$CC=\frac{V_{r}}{V_{b}}\times 100$$

where V_r_ is the volume of water retained in the saturated material (L) and V_b_ is the volume of the bottom tube (L).

According to [29], the AFP corresponds to the volume of air following saturation and free drainage. The AFP (%) can be calculated according to Equation (4):

(4) $$AFP=\frac{V_{drained}}{V_{b}}\times 100$$

where V_drained_ is the volume of water drained from the mix (L) and V_b_ is the volume of the sample (volume of the bottom tube, L).

The TEP refers to the volume of effectively filled pores of water following saturation of the material under conditions of practical use. The TEP (%) can be calculated by Equation (5).

TEP = AFP + CC (5)

The GD (kg m^−3^) is the density obtained after drying the material that was saturated according to the volume of the bottom tube that contains the sample. The GD can be found by employing Equation (6):

(6) $$GD=\frac{m_{dry}}{V_{b}}$$

where m_dry_ is the mass of the dried material (kg) and V_b_ is the volume of the sample (volume of the bottom tube, m^−3^).

The SH (%) was obtained by weighing the saturated material (m_sat_), drying it at 65 °C until it reached a constant weight (m_dry_), and employing Equation (7).

(7) $$SH=\frac{\left(m_{sat}-m_{dry}\right)}{m_{sat}}\times 100$$

The H of the material as it came from the field was obtained using a moisture balance (WPS 50SX-1).

The PS was determined according to the methodology proposed by [30]. A mass of 50 g material was applied successively to a set of six meshes (50, 16, 8, 4, 2, 0.425 mm, and bottom), shaken five times at each horizontal 90° angle, then repeated, for a total of 40 shakes. After shaking, each mesh and the remaining material was weighed, and the particle size was calculated. Moreover, the largest particles that remained in the 50 mm mesh were measured by a clipper, and the smallest particles remained at the bottom. According to [30], the APS can be calculated by the mean particle size retained in each mesh and the percentage retention relative to the total weight of the stratified sample.

All analyses of the physical properties were repeated three times. For developing the analysis, the used data consisted of the mean values of the experimental physical properties’ measurements. In total, 51 mean values were used to test the use principal components analysis (PCA) and fuzzy clustering analysis.

### 2.2. Data Analysis

The first step of data analysis was to evaluate the correlations among the physical properties of the bedding materials. The second step was to use principal components analysis (PCA) to evaluate the number of principal components (PCs) that were relevant for analysis in the subsequent steps. Fuzzy clustering analysis was then performed using the chosen PCs. Different clustering methods were tested: k-means clustering (KM), fuzzy c-means clustering (FCM), and the Gustafson–Kessel (GK) algorithms. Moreover, different numbers of clusters (2−8) were evaluated and then compared using different validation indexes. With the chosen clustering method and the number of clusters, it was possible to group the bedding materials by similar properties so as to understand their behaviour according to their physical properties.

#### 2.2.1. Principal Components Analysis (PCA)

Large datasets are increasingly widespread in different areas, and in order to interpret such datasets, methods are required for the drastic reduction in their dimensionality in an interpretable manner [31]. According to [32], PCA is used to explain the dispersion structure with a few linear combinations of the original variables. The authors in [33] state that the PCA method generates new variables as the linear uncorrelated combination out of the original variables, where the new axes or variables are called principal components, and the value of new variables are principal component scores. The number of new variables will be equal to the number of original variables. The study in [34] stated that a small number of principal components is sufficient to capture high variance among data. Thus, PCA is used to obtain a small number of linear combinations (principal components) of a set of variables that retains as much information about the original variables as possible [30,32,35].

Usually, PCA is formulated as an eigenvalue decomposition problem: each eigenvector of the sample covariance matrix of a dataset corresponds to the coefficients of a principal component [36]. It is important to use the largest eigenvalue [31]. The relative importance of a principal component may be explained by the total variance it represents; the first eigenvalue divided by the sum of all eigenvalues represents the total variation explained by the first principal component, etc. [37]. Therefore, in the present paper, the highest eigenvalues, and consequently the cumulative proportion of variance, were used to define the number of PCs to be used in the clustering analysis. For developing the PCA, the R Development Core Team computer system [38] was used.

#### 2.2.2. Fuzzy Clustering Analysis

Clustering is one of the data analysis methods used to divide a set of data objects into clusters. The objective of cluster analysis is to find the structure in a multivariate dataset that divides the data into a number of subsets [39]; thus, the idea of this analysis is to maximise interactions between elements within clusters, minimising interactions among clusters [40]. A lot of clustering methodologies exist in the literature, one of which is fuzzy clustering.

Certain algorithms are commonly used in fuzzy clustering, such as k-means clustering (KM), fuzzy c-means clustering (FCM) and Gustafson–Kessel (GK) algorithms. The research carried out in [23] highlighted that these algorithms have similar procedures, such as: (i) initial parameters and cluster centre settings; (ii) distances between data samples and cluster center calculation; and (iii) updating of a partition matrix, cluster centres, and associated parameters. This procedure is repeated until some of the criteria of termination are met.

The k-means clustering algorithm (KM) is a simple and popular analysis method [41]. The basic idea of this clustering method is to take a dataset and subdivide it into k pairwise disjoint clusters [23,41,42]. From an N × n dimensional dataset, KM allocates each data sample, x_k_ ∈ R^n^, k = 1, …, N, to one of the c clusters to minimise the intra-cluster sum of squares [23].

The fuzzy c-means (FCM) algorithm has been widely used in different fields in the literature [39,43,44,45], and [46] stated that this algorithm is the best known in fuzzy clustering. This algorithm is based on the minimization objective function. According to [39], FCM uses fuzzy memberships to characterise the extent to which each sample point belongs to a class, thereby reaching a membership matrix that represents the final clustering results.

The Gustafson–Kessel clustering algorithm (GK) differs from FCM, but according to [23], it can be considered an FCM variant. While FCM uses only cluster centres and a Euclidean distance function, GK uses an adaptive distance norm for each cluster; thus, it can detect clusters of different geometrical shapes within a dataset [47]. Therefore, the adaptative distance norm is described by the covariance matrix for each cluster, where the eigenstructure is used to identify the cluster shape [48,49]. It has been used successfully in agricultural topics, such as in studies by [23,47,50,51], in which it was stated that GK is generally more efficient than FCM due to its additional parameters. The disadvantage that GK may present is that it is more computationally costly, since the inverse and the determinant of a covariance matrix related to clusters need to be calculated at each iteration [23,49].

In the present study, three different clustering algorithms (KM, FCM, and GK) were tested in addition to different numbers of clusters for each clustering method (2−8), with the aim of reaching the best fit model to cluster the bedding materials. The results were compared in order to select the best model and the best number of clusters. Different model validation tests were used, including the partition coefficient (PC), classification entropy (CE), partition index (SC), Xie–Beni criterion (XB), and Dunn index (DI). For developing the fuzzy clustering analysis, we used MATLAB (release 2019b, The MathWorks, Inc., Natick, Massachusetts) and its Clustering Toolbox [52].

## 3. Results and Discussion

Although each physical property possesses importance for the characterization of the material, it is imperative to study the relationship among them (Table 2). The WHC was shown to be positively influenced by the SH, and the AFP was negatively influenced by the GD, CC, and BD. AFP was positively influenced by the TEP, and the BD was strongly and positively related to the GD and negatively influenced by the AFP and TEP.

The principal component analysis (PCA) generated nine PCs, with the first two PCs explaining 95% of the total variance of the bedding material physical properties data, as can be seen by the cumulative proportion (Table 3). Thus, the first two PCs can be considered significant and could be used in the clustering analysis.

The clustering analysis was carried out using the KM, FCM, and GK clustering algorithms. Different numbers of clusters were also tested. The PC, CE, SC, XB, and DI validation indexes were used to evaluate the quality of the three clustering algorithms for each number of clusters. For the PC and DI, the optimal number of clusters was at the maximum value, while for CE, SC, and XB, the optimal number of clusters was at the minimum value. In Table 4, the bold values indicate the best values of each validation index for the three tested clustering algorithms.

For the KM algorithm, the PC and CE indexes failed and resulted in no possibility of evaluation. The authors of [23] stated that PC is not applicable to KM, since it depends on the membership degree. When using the SC, XB, and DI values, the best cluster number could not be identified, since each index indicated a different number of clusters. For the FCM, the indexes indicated better results for two clusters. For GK, the SC, XB, and DI indexes indicated eight clusters as the optimal number. Table 4 shows that the GK clustering algorithm with eight clusters presented the best performance in three (SC, XB, and DI) of the five validation indexes for clustering the studied bedding materials based on their physical properties. Therefore, the GK clustering algorithm with eight clusters was chosen for division of the data.

**Figure 1:**
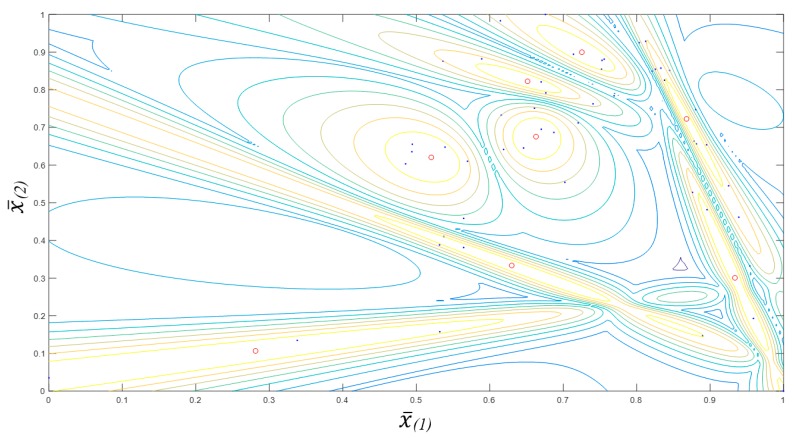
GK clusters for eight partitions; the red dots are the cluster centres and the blue dots are data samples.

As shown in Figure 1, the GK allows the formation of ellipsoids in the data space. This figure presents the soft decision boundaries provided by the GK clustering algorithm for the eight partitions; the red dots are the cluster centres and the blue dots are data samples, indicating how the data are distributed within each cluster.

Based on the cluster presented in Table 5, it can be seen that the 51 evaluated materials were divided into eight groups based on their physical properties. In some of these groups, the physical characteristic that most influenced the group is obvious. Based on these characteristics, it was possible to indicate the bedding materials that present good propensity for use.

Cluster 1 contains some of the bedding materials with the largest APS. According to [53], the larger the particle size, the lower the surface area to mass ratio. As expected, when the particle size was large, the AFP and TEP was large and the BD was low [54]. The BD is an important factor for the quantitation of the volume of products with irregular shapes; hence, the BD determines how much material can be placed at a certain site or hauled in a truck of a given size [19]. Materials in Cluster 1 had a low CC, indicating that when these materials are under saturation and drainage conditions in a given container, they have a low capacity for water retention [28]. It is important to analyse this parameter, since the CC allows comparison of different materials in a saturated situation (SH). Therefore, the materials in Cluster 1 may not be the best materials for use as bedding based on their physical properties.

Clusters 2 and 4 include some bedding materials with smaller average APS values of 4.13 and 3.61 mm, respectively. For bedding materials, small particles could pose a problem, since bedding is known to have a great impact on the dust concentration in dairy barns [55]. The study in [56] highlighted that straw and sawdust are the most commonly used types of bedding, and they can be very dusty. It was reported in [57] that it is more important to avoid bedding materials that contain a large amount of fine particles when the ventilation or fresh air exchange is restricted. Both Clusters 2 and 4 showed high average BD values of 210 and 108 kg m^−3^, respectively. Higher BD values suggest that the materials have less pore space and are more compact [58].

According to [19], higher BD values imply an increase in mass and a decrease in porosity and air volume. However, the same authors have also reported that if the material is too finely divided in compost-bedded pack systems, it is likely to be easily compacted to the point where pore space becomes inadequate to allow the sufficient movement of oxygen through the material and to maintain aerobic conditions around the particles. Thus, the lack of proper aerobic bacterial activities may hamper the function of the composting system. In this case, compost-bedded pack barn bedding materials with high BD and low AFP and TEP values may not be indicated due to poor aeration that limits oxygen uptake by microorganisms, inhibiting their growth. Conversely, for other dairy systems, such as freestalls, positive associations have been found between bacterial counts in the bedding material and those on teat ends [59,60]; hence, the occurrence of clinical mastitis is related to the total bacterial load on the teat and in the bedding material [61].

In Cluster 5, there are materials with intermediate values; however, these cannot be depicted as excellent materials, since they have a low WHC value, despite the other physical properties being moderate.

Clusters 3 and 6 can be highlighted as the best groups of materials for use as bedding for dairy cattle based on their physical properties. The main characteristic in both groups is the high WHC value; this physical property is vital to candidates for potential use as bedding for dairy cows. According to [58], high WHC values allow the bedding material to absorb water, which is a useful parameter, since it can simulate the excess urine present prior to moisture redistribution [57]. Table 2 shows that a mix of wood shavings and dry sawdust (8.80 kg kg^−1^) has the highest WHC value, followed by *Posidonia oceanica* (7.32 kg kg^−1^) and fresh sawdust (Sample 3) (5.39 kg kg^−1^).

*Posidonia oceanica* can be highlighted as a good potential material to be used as a bedding. Aside from presenting effective physical properties to be used as bedding, *Posidonia oceanica* is considered a waste material on Mediterranean beaches. According to [62], each year, beaches must be cleaned in order to remove *Posidonia oceanica* waste as it can cause odours due to its decomposition (and the subsequent appearance of insects) and create a negative visual impact, especially for tourism purposes. The same authors say that the presence of big amounts of *Posidonia oceanica* waste involves a significant financial cost to the government, which must remove thousands of tons of waste from the beaches in order to obtain quality awards. Therefore, the use of *Posidonia oceanica* as bedding material could provide a solution to this environmental and financial issue.

The main difference between Clusters 3 and 6 is that the former has smaller APS, TEP, and AFP values and, consequently, larger BD, CC, and WHC values. Both clusters have the highest SH values in comparison with the other groups, and according to Table 2, the SH is the property with the largest positive correlation with WHC. In [25], it was stated that an ideal bedding material needs to absorb moisture, dry readily, and allow animals to display natural behaviour. The same authors described the WHC as the amount of water that is absorbed and able to be stored, which is an extremely desirable property of bedding material, indicating that the materials in Clusters 3 and 6 have a good capacity to retain water. Moreover, [63] reported that the WHC value increases with increasing moisture content up to a certain level, suggesting that moisture promotes the aggregation of particles and decreases porosity.

The average BD value in Cluster 3 was larger than that in Cluster 6. According to [57], costs associated with transportation and storage are lower for denser materials. The use of a material as animal bedding also depends on its density. To determine the functional value of the bedding, the initial volume of loose materials and additions over time must be compared. This economic characteristic provides important information regarding the choice of the bedding material.

Clusters 7 and 8 show the worst average WHC values in comparison with the other groups. The lowest WHC values are presented by the eigenstructure behaviours of pine tree bark (0.61 kg kg^−1^) and the mix of fresh forest material.

The physical properties of the materials and the clusters based on these properties provide more information regarding the behaviour of different materials (those already used or novel alternatives) for use as bedding for dairy cattle. The present study draws attention to some materials that have not yet been tested in practice but have great potential for use. Further studies should be developed considering the chemical and biological characteristics of these materials. Another important point for consideration is the economic and logistic feasibility of obtaining, transporting, handling, and using these materials in different regions where there is a great demand for bedding materials.

## 4. Conclusions

The validation indexes indicate that the GK clustering algorithm with eight clusters in the dataset is the best method for clustering the bedding materials based on physical properties.

Independent of the material name, the largest influence on the cluster classification, and consequently the material’s propensity for use as a bedding material, is given by its physical properties.

*Posidonia oceanica*, which can be considered an alternative material, shows effective physical properties for use as a bedding material.

Using this clustering analysis, it is possible to understand how the physical properties of bedding materials influence their behaviour. Consequently, this type of analysis can be useful for decision making on farms that use bedding materials for dairy cattle.

## Figures and Tables

**Table 1:** Bedding materials and their country of origin.

Bedding Material	Number of Samples	Country of Origin
Pine tree bark	1	Slovenia
Barley husk	2	Slovenia
Barley straw	2	Slovenia and the Netherlands
Coniferous needle litter	1	Italy
Dried manure	1	Italy
Dry sawdust	5	Italy and from Slovenia
Flax straw	3	Slovenia
Mix of fresh forest	1	Slovenia
Fresh sawdust	3	Italy and Slovenia
Hemp straw	3	Slovenia
*Miscanthus* grass	3	Italy, the Netherlands, and Slovenia
*Posidonia oceanica*	1	Italy
Spelt husk	3	Slovenia
Triticale husk	2	Slovenia
Triticale straw	1	Italy
Wheat husk	2	Slovenia
Wheat straw	8	Italy and Slovenia
Wood chips	6	Italy, the Netherlands, and Slovenia
Wood shavings	3	Italy and Slovenia

**Table 2:** Correlations among the physical properties of the bedding materials.

Properties	WHC	AFP	GD	CC	TEP	SH	H	BD	APS
WHC	1.000								
AFP	−0.106	1.000							
GD	−0.329	−0.763	1.000						
CC	0.258	−0.961	0.665	1.000					
TEP	0.304	0.738	−0.731	−0.524	1.000				
SH	0.678	−0.122	−0.365	0.298	0.353	1.000			
H	−0.272	−0.163	0.228	0.165	−0.100	−0.136	1.000		
BD	−0.444	−0.662	0.900	0.590	−0.605	−0.290	0.542	1.000	
APS	−0.095	0.552	−0.365	−0.501	0.481	−0.259	−0.083	−0.369	1.000

Water holding capacity (WHC), air-filled porosity (AFP), global density (GD), container capacity (CC), total effective porosity (TEP), saturated humidity (SH), humidity (H), bulk density (BD), and average particle size (APS).

**Table 3:** Importance of the principal components.

Principal Components	1	2	3	4	5	6	7	8	9
Standard deviation	129.72	72.54	28.09	17.36	8.35	5.65	3.50	0.84	0.00
Proportion of variance	0.72	0.23	0.03	0.01	0.00	0.00	0.00	0.00	0.00
Cumulative proportion	0.72	0.95	0.98	1.00	1.00	1.00	1.00	1.00	1.00

**Table 4:** Validation indexes of the three fuzzy clustering algorithms for different numbers of clusters.

Number of Clusters	Validation Indexes				
	PC	CE	SC	XB	DI
**KM**					
2	1	NA	1.7471	6.6417	0.1302
3	1	NA	1.1971	4.2439	0.0667
4	1	NA	0.8542	3.1541	**0.616**
5	1	NA	0.4949	2.8345	0.1013
6	1	NA	0.4743	3.9985	0.0765
7	1	NA	0.4081	**2.3799**	5.7206 × 10^−4^
8	1	NA	**0.3609**	3.3177	0.0997
**FCM**					
2	**0.7630**	**0.3776**	2.4857	5.1551	0.0485
3	0.6817	0.5748	1.7016	2.0421	0.0622
4	0.6487	0.6679	0.9798	3.6290	0.0756
5	0.6427	0.7259	0.7563	5.5302	0.0048
6	0.6468	0.7444	0.5745	**1.9239**	0.0823
7	0.6327	0.7960	0.5257	1.9913	0.1007
8	0.6328	0.8264	**0.5041**	2.0863	**0.1016**
**GK**					
2	**0.8098**	**0.3121**	4.2196	11.4240	0.0304
3	0.7724	0.4279	1.3597	2.7344	0.0682
4	0.7769	0.4210	0.8025	2.8975	0.0615
5	0.7369	0.5221	0.6131	1.8799	0.0473
6	0.7308	0.5424	0.6657	1.6741	0.0339
7	0.7028	0.6223	0.8663	1.9032	0.0120
8	0.7270	0.5932	**0.4748**	**1.6092**	**0.0709**

Fuzzy c-means clustering algorithm (FCM); k-means clustering algorithm (KM); Gustafson–Kessel algorithm (GK); partition coefficient (PC); classification entropy (CE); partition index (SC); Xie–Beni criterion (XB); Dunn index (DI). Values in bold refer to the best performance of each validation index for each algorithm. NA means not applicable.

**Table 5:** Bedding materials clustered using the GK method based on their physical properties.

Cluster	Material	WHC	AFP	CC	TEP	SH	H	GD	BD	APS
1	Hemp straw (Sample 2)	1.60	91.92	7.43	99.35	66.44	10.12	37.55	46.93	365.79
	Hemp straw (Sample 3)	1.23	93.71	5.63	99.34	64.53	9.49	30.93	46.12	267.91
	*Miscanthus* grass (Sample 2)	2.76	89.29	7.16	96.45	67.48	28.44	34.27	26.26	141.26
	Wheat straw (Sample 1)	3.00	82.74	8.03	90.77	64.22	9.16	43.63	27.08	126.94
	**Average**	2.15 (±0.86)	89.41 (±4.80)	7.06 (±1.02)	96.48 (±4.04)	65.67 (±1.56)	14.30 (±9.44)	36.60 (±5.41)	36.60 (±11.47)	225.47 (±112.97)
2	Dry sawdust (Sample 1)	2.27	28.13	45.84	73.97	73.18	11.05	168.02	203.24	4.08
	Dry sawdust (Sample 5)	1.98	41.74	39.49	81.23	73.96	13.32	139.00	201.28	3.41
	Fresh sawdust (Sample 1)	2.14	33.22	51.84	85.06	80.61	46.35	124.78	244.82	2.51
	Fresh sawdust (Sample 2)	2.01	33.02	46.87	79.89	75.45	41.09	152.51	227.37	1.99
	Wood chips (Sample 1)	0.94	60.59	18.28	78.87	55.29	11.34	147.71	196.16	9.24
	Wood chips (Sample 6)	1.75	55.43	32.67	88.10	69.63	10.56	139.48	187.98	3.52
	**Average**	1.85 (±0.48)	42.02 (±13.24)	39.17 (±12.19)	81.19 (±4.94)	71.35 (±8.64)	22.28 (±16.71)	145.25 (±14.62)	210.14 (±21.50)	4.13 (±2.62)
3	Dried manure	4.55	46.75	32.24	78.99	83.32	14.11	64.59	71.60	11.78
	Flax straw (Sample 1)	3.25	67.44	24.01	91.45	81.99	7.87	52.62	74.64	8.32
	Fresh sawdust (Sample 3)	5.39	19.79	61.85	81.64	87.26	13.34	90.35	115.68	1.91
	Wheat straw (Sample 8)	4.12	63.40	24.85	88.24	81.27	8.61	57.27	60.34	7.47
	Wood shavings (Sample 1)	2.55	68.31	24.08	92.39	89.67	11.41	27.70	95.18	4.97
	Wood shavings (Sample 3)	8.80	35.04	52.94	87.99	84.09	9.34	99.91	60.85	5.34
	**Average**	4.78 (±2.21)	50.12 (±19.83)	36.66 (±16.59)	86.78 (±5.37)	84.6 (±3.24)	10.78 (±2.58)	65.41 (±26.32)	79.72 (±21.70)	6.63 (±3.37)
4	Dry sawdust (Sample 2)	3.73	27.78	55.38	83.16	84.03	8.02	105.29	148.69	1.40
	Dry sawdust (Sample 3)	4.52	17.10	66.02	83.11	81.46	12.20	150.48	147.31	1.50
	Flax straw (Sample 2)	2.64	56.85	30.46	87.32	78.68	10.06	82.55	116.18	4.81
	Spelt husks (Sample 1)	1.94	65.91	14.06	79.97	70.08	11.38	60.02	72.42	5.36
	Wood chips (Sample 3)	2.35	74.52	14.16	88.68	71.22	8.47	55.56	58.92	5.96
	Wood shavings (Sample 2)	3.32	53.48	34.47	87.95	78.46	9.52	94.69	104.51	2.63
	**Average**	3.08 (±0.96)	49.27 (±22.30)	35.76 (±21.30)	85.03 (±3.46)	77.32 (±5.56)	9.94 (±1.63)	91.43 (±34.76)	108.01 (±37.30)	3.61 (±2.02)
5	Barley husk (Sample 1)	1.59	68.98	18.96	87.94	69.08	10.47	84.84	119.89	3.74
	Barley husk (Sample 2)	1.65	58.64	25.01	83.65	67.87	8.80	118.36	153.40	3.42
	Coniferous needle litter	0.78	79.91	9.56	89.47	53.49	12.27	82.98	123.01	9.02
	Hemp straw (Sample 1)	2.16	53.21	26.51	79.72	75.41	10.89	86.36	123.94	4.96
	*Miscanthus* grass (Sample 1)	2.35	45.49	32.28	77.77	73.55	7.85	115.92	137.24	4.58
	*Miscanthus* grass (Sample 3)	1.82	58.06	20.24	78.30	67.15	9.52	98.91	108.30	6.63
	Spelt husks (Sample 2)	1.69	67.40	14.38	81.77	65.38	11.54	76.15	82.99	4.83
	Spelt husks (Sample 3)	1.33	57.75	18.39	76.14	65.09	11.62	98.67	135.32	3.63
	**Average**	1.67 (±0.49)	61.18 (±10.61)	20.67 (±7.16)	81.84 (±4.85)	67.13 (±6.63)	10.37 (±1.53)	95.27 (±15.53)	123.01 (±21.07)	5.1 (±1.89)
6	Barley straw (Sample 1)	5.31	81.84	8.95	90.80	78.93	8.64	23.99	16.42	172.89
	Barley straw (Sample 2)	3.02	85.67	9.74	95.41	79.11	9.84	25.67	32.60	98.04
	Flax straw (Sample 3)	1.44	83.25	3.53	86.78	68.00	10.32	16.53	23.83	231.73
	*Posidonia oceanica*	7.32	71.49	22.62	94.11	84.53	13.16	41.47	30.90	13.06
	Triticale husk (Sample 1)	2.82	86.78	7.66	94.44	79.98	10.94	19.15	27.37	10.69
	Triticale husk (Sample 2)	3.03	84.02	9.10	93.12	79.32	10.19	23.69	30.39	7.08
	Triticale straw (Sample 1)	2.90	89.85	5.63	95.49	77.05	10.02	16.76	19.60	56.55
	Wheat husk (Sample 1)	3.11	83.49	11.14	94.63	79.68	8.43	28.53	35.96	8.79
	Wheat husk (Sample 2)	2.80	81.83	10.60	92.44	78.80	9.70	28.54	36.91	7.42
	Wheat straw (Sample 3)	3.10	83.71	9.19	92.90	78.60	8.71	24.88	29.87	104.02
	Wheat straw (Sample 4)	4.83	72.66	21.35	94.01	90.30	11.12	22.70	44.71	16.58
	Wheat straw (Sample 5)	3.00	86.42	6.59	93.01	77.11	9.22	19.50	21.97	103.28
	Wheat straw (Sample 6)	4.07	81.60	12.48	94.08	80.75	10.93	29.58	29.88	32.02
	Wheat straw (Sample 7)	3.53	85.56	9.88	95.44	81.06	8.52	22.91	28.24	205.81
	**Average**	3.59 (±1.43)	82.73 (±5.05)	10.61 (±5.34)	93.33 (±2.29)	79.52 (±4.74)	9.98 (±1.30)	24.56 (±6.39)	29.19 (±7.35)	76.28 (±78.71)
7	Wheat straw (Sample 2)	1.64	80.98	3.23	84.21	29.29	9.99	77.94	19.72	214.90
	Wood chips (Sample 2)	0.83	59.71	18.30	78.01	53.30	17.95	160.34	222.27	11.94
	Wood chips (Sample 4)	1.02	55.18	20.29	75.46	53.65	8.85	175.79	200.27	22.09
	Wood chips (Sample 5)	0.83	62.65	16.76	79.41	52.79	19.77	149.67	204.43	24.95
	**Average**	1.08 (±0.39)	64.63 (±11.33)	14.64 (±7.74)	79.27 (±3.68)	47.26 (±11.98)	14.14 (±5.52)	140.94 (±43.34)	161.67 (±95.12)	68.47 (±97.78)
8	Pine tree bark	0.61	65.75	17.73	83.48	60.09	57.75	130.59	285.43	69.78
	Dry sawdust (Sample 4)	1.27	5.93	67.90	73.83	61.91	8.02	418.54	532.93	2.89
	Mix of fresh forest	0.70	56.27	31.70	87.98	67.40	63.81	153.10	453.10	7.93
	**Average**	0.86 (±0.36)	42.65 (±32.15)	39.11 (±25.89)	81.76 (±7.23)	63.13 (±3.81)	43.19 (±30.61)	234.07 (±160.15)	423.82 (±126.32)	26.87 (±37.25)

Water holding capacity (WHC) (kg.kg^−1^); air-filled porosity (AFP) (%); container capacity (CC) (%); total effective porosity (TEP) (%); saturated humidity (SH) (%); humidity (H) (%); global density (GD) (kg m^−3^); bulk density (BD) (kg m^−3^); average particle size (APS) (mm).
